# Time series analysis of demographic and temporal trends of tuberculosis in Singapore

**DOI:** 10.1186/1471-2458-14-1121

**Published:** 2014-10-31

**Authors:** Win Wah, Sourav Das, Arul Earnest, Leo Kang Yang Lim, Cynthia Bin Eng Chee, Alex Richard Cook, Yee Tang Wang, Khin Mar Kyi Win, Marcus Eng Hock Ong, Li Yang Hsu

**Affiliations:** Centre for Infectious Disease Epidemiology & Research, Saw Swee Hock School of Public Health, National University of Singapore and NUHS, Singapore, Singapore; Centre for Quantitative Medicine, Duke NUS Graduate Medical School, Singapore, Singapore; Singapore Tuberculosis Elimination Programme, Tan Tock Seng Hospital, Novena, Singapore; Department of Emergency Medicine, Singapore General Hospital, Singapore, Singapore

**Keywords:** Time-series analysis, Tuberculosis

## Abstract

**Background:**

Singapore is an intermediate tuberculosis (TB) incidence country, with a recent rise in TB incidence from 2008, after a fall in incidence since 1998. This study identified population characteristics that were associated with the recent increase in TB cases, and built a predictive model of TB risk in Singapore.

**Methods:**

Retrospective time series analysis was used to study TB notification data collected from 1995 to 2011 from the Singapore Tuberculosis Elimination Program (STEP) registry. A predictive model was developed based on the data collected from 1995 to 2010 and validated using the data collected in 2011.

**Results:**

There was a significant difference in demographic characteristics between resident and non-resident TB cases. TB risk was higher in non-residents than in residents throughout the period. We found no significant association between demographic and macro-economic factors and annual incidence of TB with or without adjusting for the population-at-risk. Despite growing non-resident population, there was a significant decrease in the non-resident TB risk (*p* < 0.0001). However, there was no evidence of trend in the resident TB risk over this time period, though differences between different demographic groups were apparent with ethnic minorities experiencing higher incidence rates.

**Conclusion:**

The study found that despite an increasing size of non-resident population, TB risk among non-residents was decreasing at a rate of about 3% per year. There was an apparent seasonality in the TB reporting.

**Electronic supplementary material:**

The online version of this article (doi:10.1186/1471-2458-14-1121) contains supplementary material, which is available to authorized users.

## Background

Tuberculosis (TB) is a leading cause of death globally [1]. Approximately 5% of those infected progress to active disease within the first two years, but the majority experience a state of prolonged latency with a life-time risk of 10-15% eventually progressing to active disease [2]. The risk of progression is affected by immune status and is much higher among immune-compromised individuals [2]. The situation is complicated further by the spread of multidrug-resistant (MDR) TB [3], a particular problem in South East Asia.

Since the World Health Organization (WHO) declared TB to be a global health emergency in 1993 [4], there has been a substantial progress on many indicators [3]. The most significant achievement was that global TB mortality reduced by 45% in 2012 [3]. However, the rate of decrease of global TB incidence (2% per annum) remains quite low [3]. In this context, the study of TB prevalence in Singapore is important for the following two reasons. As an economically developed city state (GDP per capita $52,000) [5], Singapore, lies on an important maritime transit route, is a transit hub for travel in Asia and Asia-Pacific and a significant global destination of investment and labour. However, it is also a part of the region that accounts for 29% of global TB incidence [3]. Contrasting the global trends of most developed nations, Singapore has witnessed a resurgence of TB incidence since 2008.

TB incidence rates among Singapore residents declined from 300 (per 100,000 population) in the early 1960s to 106 (per 100,000 population) in the mid-1980s [6, 7]. In 1997, the Singapore Tuberculosis Elimination Programme (STEP) was launched to address a decade-long stagnation in TB rates at 50–55 (per 100,000 population) between 1987 to 1997. Thereafter, TB incidence rates among Singapore residents declined from 58 (per 100,000 population) in 1998 to an historic low of 35 (per 100,000 population) in 2006 and 2007. However, the TB incidence rates began to rise around 39 per 100,000 population since 2008. The reasons for this surge remain unclear since there has been no association study on the influence of the demographic characteristics on TB in Singapore.

In this research, our aim was to assess temporal dynamics in TB incidence in Singapore at both short (monthly) and large (annual) scales, and to investigate the association of several population level demographic factors with annual TB incidence. For the annual analysis. the primary methodology we used was the standard autoregressive integrated moving average (ARIMA) regression model [8]. Additionally, using monthly data from 1995–2011, we assessed whether the burgeoning non-resident population led to an increase in TB risk in Singapore. We proposed using the seasonal autoregressive moving average (SARIMA), ARIMA models with periodic components, to predict the temporal trends of the more volatile monthly TB risk among residents and non-residents in Singapore and detect seasonality.

## Methods

### Study setting

Singapore is a city-state in the middle of the Malay archipelago approximately 100 Km from the equator. Its population of 5.3 million (2013) has grown rapidly over the last decade due to the influx of foreigners who now constitute approximately 28% of the total population, comprising 3.8 million residents (citizens and permanent residents) and 1.5 million non-residents (i.e. those who hold long-term visit or work passes) [5]. The proportion of the latter group has increased markedly since 2005, with most migrants coming from South and East Asian countries.

### Data

This study retrospectively analyzed all newly laboratory-confirmed TB cases reported to Singapore Tuberculosis Elimination Program (STEP) registry from 1995 to 2011. Laboratory confirmation was based on positive *M. tuberculosis* culture. Under the Infectious Disease Act in Singapore, all suspected and confirmed TB cases are to be notified to the STEP registry of the TB Control Unit (TBCU) within 72 hours of starting TB treatment and/or laboratory confirmed results. The TBCU is the national unit for treatment of TB patients, contact investigation, preventive therapy, and educational training of health care workers in Singapore.

Population data were obtained from Singapore Department of Statistics and Ministry of Health [9, 10].

### Statistics and analysis

#### Annual TB cases analysis: 1995–2011

We fitted time series (ARIMA) regression models for the yearly time series counts of TB cases to establish whether a relationship exists between population characteristics and yearly TB cases over time. We used the standard Box Jenkins approach [11] to fit the ARIMA models, characterized by 3 main parameters: an autoregressive (AR) term, indicating the strength of relationship between successive years’ incidence, a moving average (MA) term, that checks for the dependence of yearly incidence on current and past model residuals, and a differencing term, that is usually applied to make the data stationary, when the time series has a long term trend [11]. Further as the annual TB time series exhibited change of variance over the time horizon, yearly notifications were log transformed, which had the effect of standardizing the scale of the error terms.

Following stand and procedure, sample autocorrelation (ACF) and partial autocorrelation function (PACF) plots were used to identify the order of the MA and AR terms included in the ARIMA model [12, 13]. Specifically, if we observe statistically significant autocorrelation in the sample ACF plot at *q* time lags and significant sample PACF at *p* lags then, an ARIMA model with *p*-AR components and *q*-MA components is proposed. The final model (order) selection is made using a combination of goodness of fit and Akaike’s Information Criterion (AIC). Here we the determined the goodness of fit using the residual autocorrelation function to ensure that no additional autocorrelation was present in each fitted model, and models were compared using Akaike’s Information Criterion (AIC). The model with the lowest value of the AIC was selected to analyze yearly TB cases. We developed separate univariate models, each encompassing a different lag period controlling the effect of potential predictor (from lag 0, that is the current year, to the immediate preceding 4 years). Population variables included numbers of visitors’ arrivals, the sizes of the elderly population, the non-resident population, and the resident population broken down by ethnicity (Chinese, Indian, Malay and Other ethnicity), Gross Domestic Product (GDP) rates, population density and HIV notification rates. Population density was the mid-year population estimate divided by the country’s area (square kilometers) which grew during the study period due to land reclamation. We also adjusted for the population-at-risk (resident or non-resident population) as the denominator for demographic variables that did not already account for them, such as GDP, HIV notifications and visitor’s arrivals.

Mid-year population estimates from the Department of Statistics were used to generate crude incidence and age-standardized incidence rates [5]. In the direct method of standardization, all age-standardized incidence rates were derived by applying the category-specific incidence rates of each population to the standard world population [14].

#### Monthly TB risk analysis: 1996–2011

The limitation of the above annual analysis is that the available length of annual time series is small and so the analysis is underpowered we therefore supplement it with a shorter, monthly time step. However we have sufficient monthly temporal observations. In this section we describe the method used to investigate monthly temporal risk of TB.

The TB risk among resident or non-resident population was defined as Y_t_ = (c_t_/n_t_) × 100 000, where c_t_ denotes the monthly count of TB reported cases (residents or non-residents) and n_t_ is assumed to be population-at-risk (number of resident or non-resident population) in month *t*. This definition takes into account the dynamics of the population size in explaining TB incidence. Population figures were derived from annual population data [5].

To investigate the monthly time series of TB risk we use classical time series decomposition [11] that is conceptually,


In our models we have used linear and cosine components to model the trend and cyclic components while seasonality and irregular components are modelled using SARIMA models (see [12]).

To account for the observed periodicity in the time series, Y_t_, we introduced a combination of cosine components. This is a well known approach for addressing deterministic periodic effects in a time series data (see for example [Ch 5, 30] [15]) , and Wiener and Khintchine [13] have shown, in a seminal result, that a stationary time series can be completely written as a combination of sine and cosine components and error. From an application point of view this technique of modelling the trend and cyclical components can be perceived as a regression of the data Y_t_ on periodic covariates, which are functions of time, while the stochastic dependence of the data, on its past including seasonality, is modelled by fitting SARIMA models to the residuals of this regression.

Plots of incidence over time showed volatility in the yearly and monthly TB-reporting indicating a possible heteroskedasticity of variance. We converted the raw data to its natural logarithm to stabilize the variance of the time series and improve the estimation.

Explicit formulae for modelling the trend of resident and non-resident monthly TB risk are given below. We use the subscripts “R” and “NR” to indicate model components for residents and non-residents respectively.

### Resident model



### Non-resident model



In each model *β*_*0*_ is the intercept term and *β*_*1*_ is the long term linear rate of change of TB risk per month. The principal reason for proposing the linear temporal component is to assess if monthly TB risk overall follows an increasing or decreasing trend, *β*_*k*_ are the coefficients of the *k*^*th*^ time period *T*_*k*_. All periods T_k_ are greater than 1 year. Based on sample autocorrelation and periodogram analysis of *log(Y*_*t*_*)* we proposed periods, *T*_*k*_. The corresponding coefficients β are then estimated and tested for statistical significance, using the method of least squares. Specific details on *k* and the coefficients *β* for each model are given in the Results section and Table 1. The ‘residual’ correlated noise, *e*_*t*_*,* were modelled using SARIMA models. Details of SARIMA models are given in Table 2. Initial investigation suggested that the models were both appropriate and parsimonious.

**Table 1:** **Linear model coefficient estimates of TB risk among residents and non-residents (1995–2011)**

Lag 0				
**Non-resident model**	**Estimate**	**Lower confidence interval (2.5%)**	**Upper confidence interval (97.5%)**	***p*** **value**
Intercept	2.26	2.19	2.32	< 0.001*
t	−0.003	−0.0032	−0.0019	< 0.001*
cos(2*(pi/180)*t)	0.233	0.186	0.279	< 0.001*
Adjusted R-square	0.47			
**Resident model**	**Estimate**	**Lower confidence interval (2.5%)**	**Upper confidence interval (97.5%)**	***p*** **value**
Intercept	0.639	0.573	0.706	<0.001*
t	−0.0002	−0.0008	−0.0005	0.602
cos(2*(pi/50)*t)	−0.063	−0.109	−0.017	0.008*
cos(2*(pi/100)*t)	−0.068	−0.119	−0.017	0.009*
cos(2*(pi/17)*t)	−0.046	−0.092	0.0001	0.051
cos(2*(pi/180)*t)	0.302	0.254	0.351	<0.001*
Adjusted R-square	0.53			
**Lag 4**				
**Non-resident model**	**Estimate**	**Lower confidence interval (2.5%)**	**Upper confidence interval (97.5%)**	***p*** **value**
Intercept	2.579	2.51	2.65	< 0.001*
t	−0.003	−0.0038	−0.0025	< 0.001*
cos(2*(pi/180)*t)	0.478	0.429	0.527	< 0.001*
Adjusted R-square	0.72			
**Resident model**	**Estimate**	**Lower confidence interval (2.5%)**	**Upper confidence interval (97.5%)**	***p*** **value**
Intercept	0.708	0.642	0.774	<0.001*
t	−0.0009	0.0004	−0.802	0.424
cos(2*(pi/50)*t)	−0.064	−0.111	−0.018	0.007*
cos(2*(pi/100)*t)	−0.075	−0.125	−0.024	0.004*
cos(2*(pi/17)*t)	−0.046	−0.092	0.00007	0.050
cos(2*(pi/180)*t)	0.314	0.266	0.362	<0.001*
Adjusted R-square	0.550			

*; Statistically significant (*p* < 0.05).

**Table 2:** **Time series model selection criteria for TB risk residuals**

	Lag 0		Lag 4	
Model (Non-residents)	AIC	MSPE	AIC	MSPE
AR(2)	−42.79	5.01	−37.6	5.13
MA(2)	−42.88	5.06	−36.39	5.09
ARMA(2,2)	−46.08	4.5	−35.42	4.8
**SARIMA(1,0,0)(2,0,0)** [14]*	−51.1^#^	4.54^#^	−46.49^#^	4.28^#^
**Model (Residents)**	**AIC**	**MSPE**	**AIC**	**MSPE**
AR(2)	−42.66	0.85	−51.67	1.56
MA(3)	−39.05	0.86	−51.35	1.57
ARMA(2,2)	−39.17	0.87	−48.36	1.57
**SARIMA(1,0,0)(2,0,0)** [14]*	−49.18^#^	0.77^#^	−59.42^#^	1.48^#^

AIC; Akaike information criterion, MSPE; Mean squared prediction error, AR; Autoregressive, MA; Moving average, ARMA; Autoregressive moving average, SARIMA; Seasonal Autoregressive Integrated Moving Average,

*; A SARIMA model for residual, with 12 month seasonality, seasonal autoregressive component of order 2 and linear autoregressive component of order 1.

^#^; lower AIC and MSPE values indicate better fit of the model.

The long latency of TB is frequently observed as a major challenge in constructing epidemiological models for TB [16]. Therefore, we fitted similar linear models for TB risk to adjust for different latent periods of lag 0–4 years by changing the susceptible population n_t_, denominator of *Y*_*t*_, to be the population size in the appropriately lagged year. For example, to account for a latent period of 1 year, we divided TB count for a particular time point by the population at risk during previous year.

The prediction models were developed using monthly reported TB data between 1996 and 2010 and validated by predicting the data for 2011.

The goodness of fit was examined using AIC, Mean Squared Prediction Error (MSPE) and residual autocorrelation function. A lower AIC value and MSPE indicate a better fit of the model. The best-fitting model was used to build the subsequent predictive model. The prediction models were fit using the generic function auto.arima( ) in the forecast package of R [17]. Approval was obtained from the institutional review board (DSRB 2009/00232) to conduct this study.

## Results

**Figure 1:**
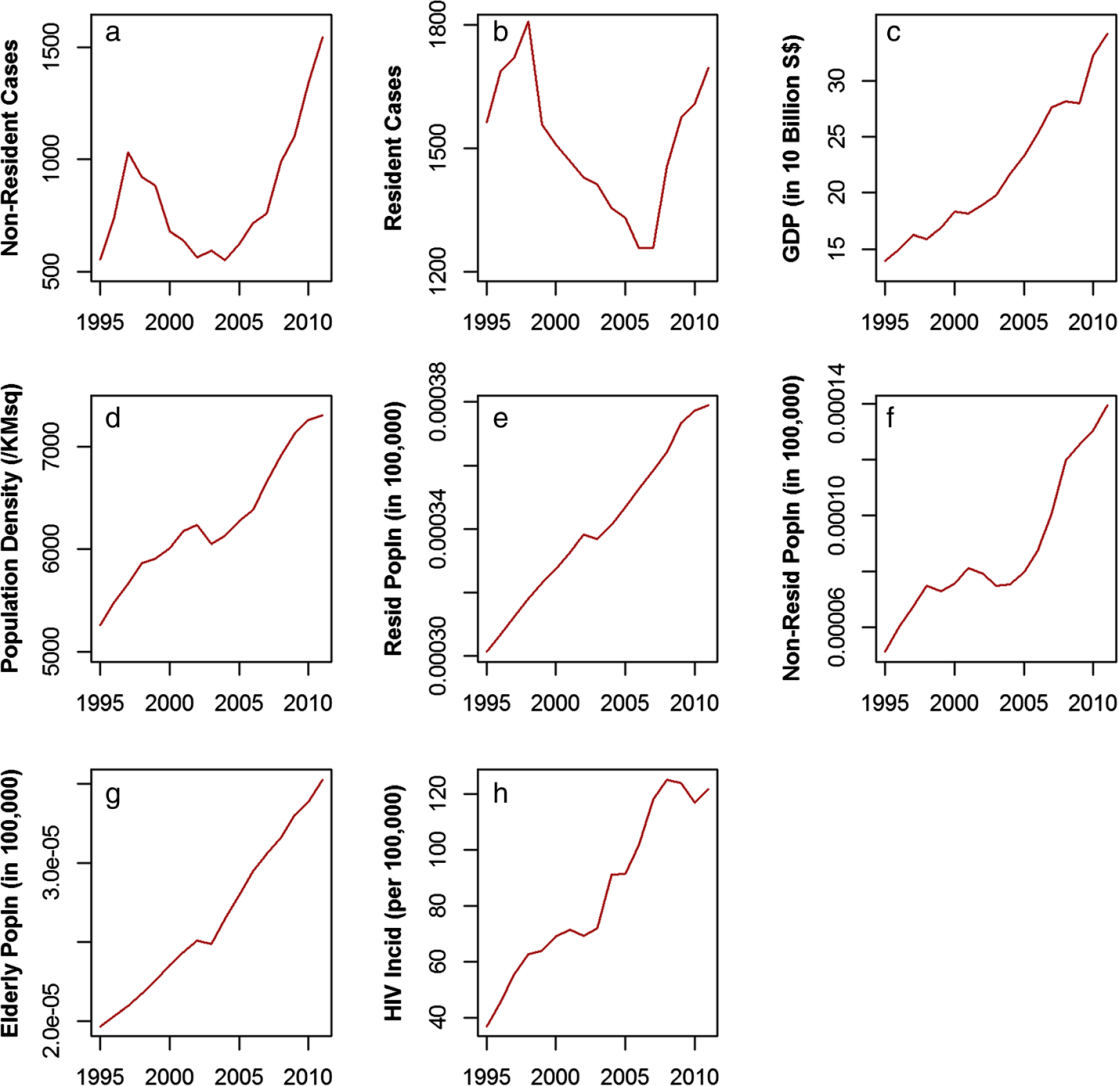
**Non-resident and resident TB cases with population characteristics; (a) Non-resident TB cases (b) Resident TB cases (c) GDP (in 10000$) (d) Population density (per sqkm) (e) Resident population (in 100,000) (f) Non-resident population (in 100,000) (g) Elderly population (Age over 65) (in 100,000) (h) HIV incidence rate (Per 100,000 population).**

There were 40,046 laboratory-confirmed TB cases reported to the STEP registry between 1995 and 2011. Table 3 shows the demographic characteristics of TB cases by residential status. Among both residents and non-residents, most TB cases were aged 15–64 years old. Compared to resident TB cases, non-resident cases were more likely to be younger, belong to other (i.e. not belonging to any of the three main ethnic groups in Singapore, Chinese, Indian and Malay) ethnic groups and to be in prison. As residents and non-residents have substantially different demographic features and health issues, we did separate time series analyses for the two groups. Figure 1 shows the increasing trend of resident and non-resident TB cases corresponding to increasing population size and density. The proportion of non-resident TB cases contributing to the total case burden increased from 25.5% in 1995 and 28.9% in 2004 to 47.7% in 2011.

**Table 3:** **Demographic characteristic of TB cases by resident status**

Characteristics	Patients (n = 40,046)	Residents (n = 25680) (64.1%)	Non-residents (n = 14210) (35.5%)	***p***value
Median age (Interquartile range), years	39,886	53.83 (39.17-68.17)	30 (25.17-38.83)	<0.001*
**Age groups**				
<14	401	306 (1.2)	95 (0.7)	<0.001*
15-64	30957	17544 (68.4)	13413 (94.5)	
>65	8493	7813 (30.4)	680 (4.8)	
**Ethnicity**				
Chinese	23256	19113 (74.4)	4143 (29.2)	<0.001*
Indian	3405	1470 (5.7)	1935 (13.6)	
Malay	5391	4496 (17.5)	895 (6.3)	
Other	7830	601 (2.3)	7229 (50.9)	
**Prison**				
Prison cases	397	201 (0.8)	196 (1.4)	<0.001*
Civilian cases	39493	25479 (99.2)	14014 (98.6)	

*; Statistically significant (p<0.05).

### Predictors of annual TB cases

On the basis of parameter estimation and goodness of fit test statistics, plausible ARIMA models were used for time series regression analysis. For residents, an ARIMA (2, 0, 1) model was selected whereas for non-residents, an ARIMA (1, 1, 1) was preferred. We found no significant association between demographic factors and annual incidence of TB with or without adjustment of population-at-risk.

### Annual TB risk analysis

**Figure 2:**
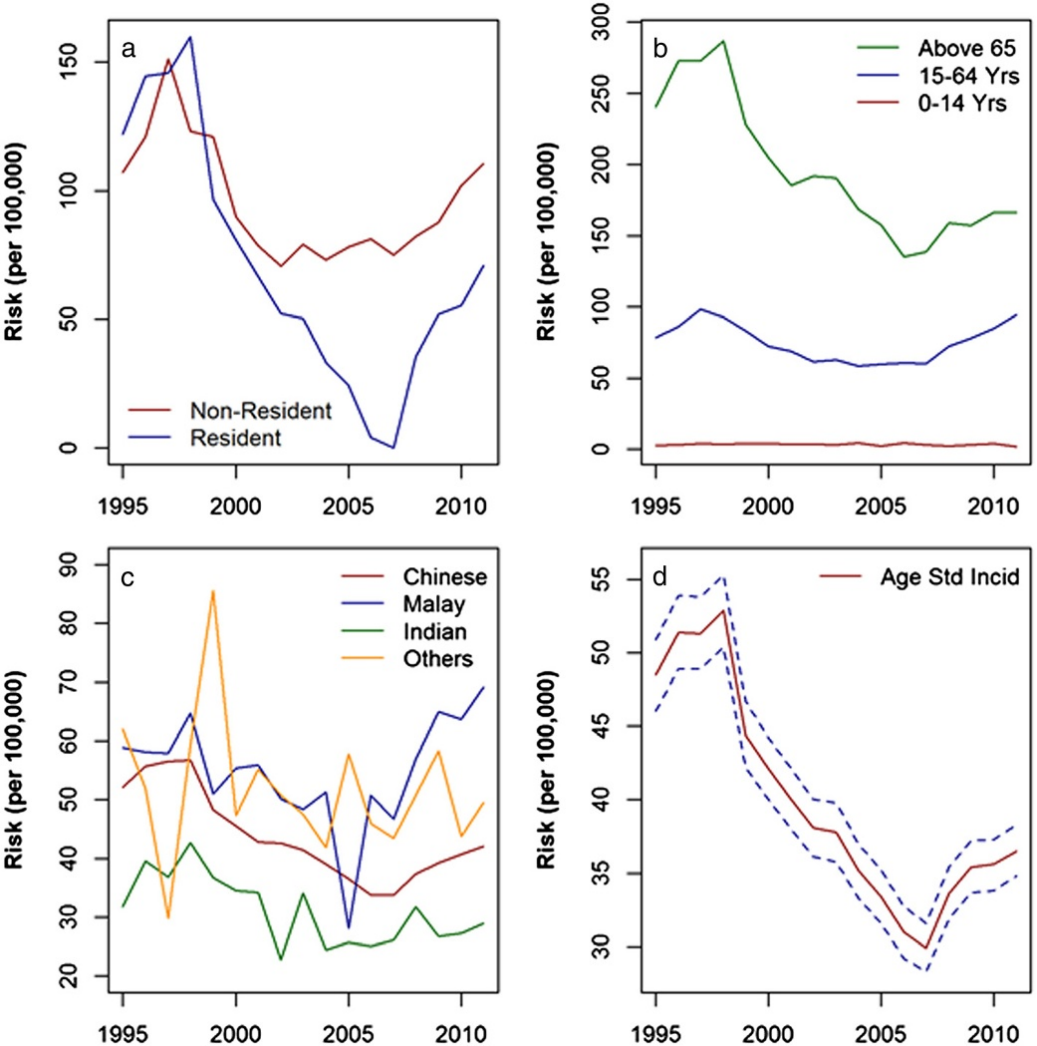
**Crude incidence rates by resident status, age groups and ethnicity and age-standardized incidence rates; (a) Comparative risk plot of Tuberculosis among non-resident and resident communities in Singapore (b) Age-specific incidence rates of Tuberculosis (Residents) (c) Ethnicity-specific incidence rates of Tuberculosis (Residents) (d) Age-standardized incidence rates (Residents); dashed lines are 95% confidence intervals.**

TB incidence rates were consistently higher among non-residents than residents over the period studied (Figure 2a). Among residents, the TB incidence rate was higher among those aged ≥65 years and who belong to Malay and Other ethnic groups (Figure 2b,c). Age-standardized incidence rates (ASIR) among residents were significantly lower in 2011 (ASIR: 36.5 per 100,000 population, 95% CI = 34.8-38.3 per 100,000 population) by 24% compared with 1995 (ASIR: 48.5 per 100,000 population, 95% CI = 46-50.9 per 100,000 population) (Figure 2d). Despite a drop between 2004 and 2007, the ASIR has been increasing since 2008. Compared with the 2007 ASIR (29.9 per 100,000 population, 95% CI = 28.3-31.6 per 100,000 population), there was a 22% increase by 2011 (36.5 per 100,000 population, 95% CI = 34.8-38.3 per 100,000 population). When stratified by ethnic groups among residents, Malay and Other ethnicities had significantly higher ASIR over years (Additional file 1: Figure S1).

### Monthly TB risk prediction

We noted that the TB incidence rates continued to be higher among non-residents than residents. The annual time series analysis also indicated that TB incidence among non-residents continued to grow with an increasing population size. However, from a public health perspective, it is important to assess the dynamics of TB incidence relative to the growth in population size (TB risk).

This study found that TB risk among the non-resident population was significantly linearly decreasing at a rate of 3% per year (monthly linear estimate = −0.3%, 95% CI = −0.3%, -1.9%, *p* < 0.0001) (Table 1).

However, we found no increasing or decreasing linear trend in the resident TB risk. Both resident and non-resident TB risk time series had significant long-term deterministic periodicities. We modelled these patterns using standard trigonometric components (detailed in Table 1). For residents we observe that the monthly TB risk time series has periodicities of approximately 18 months, 4 years, 8 years. While for the non-resident group we observe that periodicity coincides with the length of the time series. Additional file 2: Figure S2 shows the fitted curves superimposed on the resident and non-resident TB risk. From a purely epidemiology point of view, we would not wish to ascribe too much importance to the particular values of periodicity beyond its contribution to goodness-of-fit of the model. However the statistical significance of the periodic components underscore that monthly risk (and hence the monthly reported cases) have multiple long term periodicities and further research is needed to understand if there are socio-economic factors influencing these periodic effects.

The linear models with suspected latent periods of 0–4 years among residents and non-residents were found to be similar. In particular, for each lagged model, the rate of change of TB risk, Y_t_, continued to be statistically significantly negative among all lags of latent period. Due to the similarity of the above lagged linear models, we reported the analyses with lag 0 and 4 (Table 1). Next, we investigated the autocorrelation among monthly residuals of the above linear models. The sample autocorrelation plots for the residuals of linear models for both resident and non-resident TB risk show a slow decaying periodic nature with significant autocorrelation at lags of multiples of 12 months (Additional file 3: Figure S3 and Additional file 4: Figure S4). On average, for both Residents and Non-Residents the peak of TB risk is observed in the month of July with relatively more cases are reported in March, July and October, though we would like to point out that this seasonal difference is not substantial. These observations in a time series warrant the use of SARIMA models [13].

Based on AIC and MSPE values, we selected SARIMA models to be the best fit for monthly TB risk of residents and non-residents (Table 2).

Additional file 5: Figures S5 and Additional file 6: Figure S6 show the sample autocorrelation and partial autocorrelation of residuals of the final SARIMA models. Sample autocorrelation of residuals are within the bounds of 95% confidence interval with no significant autocorrelation between residuals at different lag times, and the models passed the Box-Ljung test (*p* = 0.63) on auto-correlation of the final residuals and Kolmogorov-Smirnov test for normality (*p* = 0.58). Thus, we selected an additive combination of a deterministic linear model and a stochastic SARIMA for resident and non-resident TB risk. This analysis detected a 12-month stochastic seasonality and a long term deterministic periodicity in the reporting patterns of resident and non-resident TB risk.

### Note

Different models and modeling techniques can be used for making inference on the same time series data. The periodic nature of sample autocorrelation function plots for residuals of the trend adjusted time series of TB risk for residents as well as non-residents depicted in Additional file 3: Figure S3 and Additional file 4: Figure S4 indicate that one could also use Generalized Autoregressive Moving Average models [18].

## Discussion

Although the TB incidence rate in Singapore is lower than most other Asian countries, it is higher than most other developed countries [3]. We observed that TB incidence rates were higher among the elderly, Malay, the ‘Other’ ethnic group (a mix of other Asian ethnicities, Europeans and those of mixed racial heritage) and non-residents. In Singapore, the resident elderly population had almost doubled by 2011 compared to 1995 [9] and the higher TB incidence in the elderly group could partly explain the increase in TB incidence in the resident population since 2008. Among all resident ethnic groups, the Malay group had the highest increase in ASIR. A previous study showed that this ethnic group in Singapore has clinical and socio-economic vulnerability to TB infection and transmission [19].

Although HIV notification rates have been rising in Singapore, the increasing trend of HIV did not have a significant influence on yearly TB cases. This is possibly because the number of HIV cases is low especially in comparison to the overall TB burden in Singapore [20]. In addition, non-residents applying for work permits in Singapore are screened for HIV on arrival and are deported if they subsequently are diagnosed to be infected, limiting the impact of HIV on TB in the non-resident population.

Since around 2005, liberalization of immigration policy resulted in a marked increase in population and economic growth with a rapid influx of foreign-born immigrants in Singapore. The non-resident population of Singapore has grown to about 37% of the country’s total labor force in 2012 [21]. Majority of immigrants come from high incidence TB countries such as India and China. An increasing trend of non-resident TB cases contributing to the overall proportion of TB cases over the years could suggest that mass immigration from high TB incidence countries is increasingly contributing to burden of TB in recent years in Singapore, similar to other low-incidence countries such as the US, Canada and the UK [22]. Given plausible interactions between residents and non-residents, TB infections in the non-resident population might have led to a subsequent transmission to residents, and a recent DNA finger-printing study in Singapore also suggested a cross-transmission between residents and non-residents [23]. Consistent with this hypothesis, the increasing trend of TB incidence among residents since 2008 seemed to follow a similar rise among non-residents after 2005. However, our study did not find a direct influence of increasing number of non-resident population on resident TB cases. Importantly, when adjusted for population-at-risk, the TB risk among non-resident population was found to be decreasing at a rate of about 3% per year despite the burgeoning size of the non-resident population.

We found both short term seasonal and long term periodic variations in TB reporting. Seasonal variation has been reported in the countries with distinct climatic seasons [24–28], but its presence in Singapore is curious as this city state is located very close to equator and has little climatic variability across the year. This study highlights a need for further studies to identify the factors explaining the seasonality of TB reporting patterns in Singapore. This study has shown that SARIMA model could be useful in the short-term prediction of TB in Singapore.

Under the implementation of National Tuberculosis Program in Singapore, non-residents can also access free DOTS treatment since 2010 in Singapore [29]. This could be a plausible factor behind a decreasing trend of TB risk among non-residents. Additionally, it could also be explained due to a much greater influx of employment pass holders (professionals and white collar workers) than work permit holders (manual laborers and domestic workers) in the recent years [30]. Findings from this study may permit better understanding of the underlying time-series dynamics of TB risk among different demographic groups and review TB control measures at the national scale in Singapore. In ongoing research, we have identified that TB even within a city state of Singapore is a heterogeneous multilevel event influenced by several demographic, socio-economic, spatio-temporal and environmental factors. Thus, strategies for intervention need to be more specific for any demographic group and not based solely on overall incidence.

A clear strength of the current study is the completeness and consistency of the data: Singapore, as a city that is also a country, has a clearly demarcated borderline and population catchment, while legislation means that any case of TB has to be notified by the attending clinician to the ministry of health. Over the course of the study period, all such notifications have been shared with the national TB registry that has collated these data consistently. Another strength is the rapidness of changes to the population, which potentially would allow large effects of demographics to be seen.

However there are also limitations. These could be broadly classified as either systemic or epidemiological. A major limitation is that the study period is relatively short for annual analysis. The implication of this is that there is limited power to detect smaller demographic effects, and especially to identify independent effects of demographic changes at an annual level, given that many indicators have risen in tandem with each other. The monthly analysis is an attempt to identify effects at other temporal scales. While this limits the ability to relate TB incidence to longer scale changes, such as demographic indices for which only yearly data are available, it does permit estimation of secular changes and within-year changes, modelled as linear and sinusoidal terms, respectively. Use of monthly data naturally increases the data size and makes a more powerful analysis than the yearly analysis by itself.

First, the ecological nature of the study limited the ability to draw conclusions at the individual level due to ecological bias. A further difficulty is the complicated epidemiology of TB, with a mixture of immediate onset of symptoms in some cases and a long latent period in others. This means that any demographic changes with a genuine effect on TB infection may have their influence dispersed over time, further lowering the power. Another weakness is the absence of socio-economic and demographic information on non-residents, beyond their total numbers, as these are not published by the government. Additionally, non-resident TB cases considerd in this study included both long-stay (dependent pass, work pass, student pass and long term social visit pass) and short-stay (short-term social visit pass with a maximum of 89 days) non-residents. We expected short-stay non-residents might have contributed to the overall non-resident TB cases to a small degree and exclusion of these cases might not have affected our results to a large extent. Lastly, as we could only obtain reliable data from 1995, we could not reliably assess the impact of STEP (which was formed in 1997) itself. Since the data are from a passive surveillance system, the possible biases in disease reporting and potential underreporting of TB cases might influence the precision of the analysis. However, laboratory-confirmed tuberculosis is notifiable by law in Singapore, and is moreover notified by both the laboratory and the physician-in-charge. There was also no major difference in data collection and extraction procedures used throughout the study period, hence it is very unlikely that there were significant errors. Nonetheless, a significant proportion of TB cases are culture-negative, and discarding these cases may have affected the analyses. Additionally, careful assessment of the accuracy and comprehensiveness is required when interpreting the result of the analysis since TB notifications may not be considered as a direct measure of TB incidence [31]. Because of the long latency of TB, a longer period of time is required to determine the impact of increasing non-resident TB incidence on the resident population. Future molecular epidemiological studies are needed to differentiate recent infection or reactivation of TB between resident and non-resident population over time.

## Conclusion

The study found that despite an increasing size of non-resident population, TB risk among non-residents was decreasing at a rate of about 3% per year. There was an apparent seasonality in the TB reporting patterns and SARIMA model could be useful in predicting the short-term trend of TB risk.

## Electronic supplementary material

Additional file 1: Figure S1: Age-standardized incidence rate of tuberculosis, stratified by ethnicity. (TIFF 1010 KB)

Additional file 2: Figure S2: Trend model of monthly TB risk (a) residents (b) non-residents. (TIFF 882 KB)

Additional file 3: Figure S3: Autocorrelation (ACF) and partial autocorrelation (PACF) plots of fitted residuals (Residents). (TIFF 859 KB)

Additional file 4: Figure S4: Autocorrelation (ACF) and partial autocorrelation (PACF) plots of fitted residuals (Non-residents). (TIFF 858 KB)

Additional file 5: Figure S5: Autocorrelation and partial autocorrelation of final SARIMA model residuals (Residents). (TIFF 888 KB)

Additional file 6: Figure S6: Autocorrelation and partial autocorrelation of final SARIMA model residuals (Non-residents). (TIFF 898 KB)

## Abbreviations

TB: Tuberculosis
ARIMA: Autoregressive integrated moving average
STEP: Singapore tuberculosis elimination program
TBCU: TB control unit
GDP: Gross domestic product
ACF: Autocorrelation function
PACF: Partial autocorrelation function
AIC: Akaike information criterion
DOTS: Directly observed treatment short course
MSPE: Mean square prediction error
SARIMA: Seasonal autoregressive integrated moving average
ASIR: Age-standardized incidence rates
AR: Autoregressive
MA: Moving average
D: Differencing.
