# Supplementary figures and images for: Time series analysis of demographic and temporal trends of tuberculosis in Singapore
Source: BMC Public Health. 2014 Oct 31;14:1121. doi: 10.1186/1471-2458-14-1121 (PMC4230736; doi:10.1186/1471-2458-14-1121)

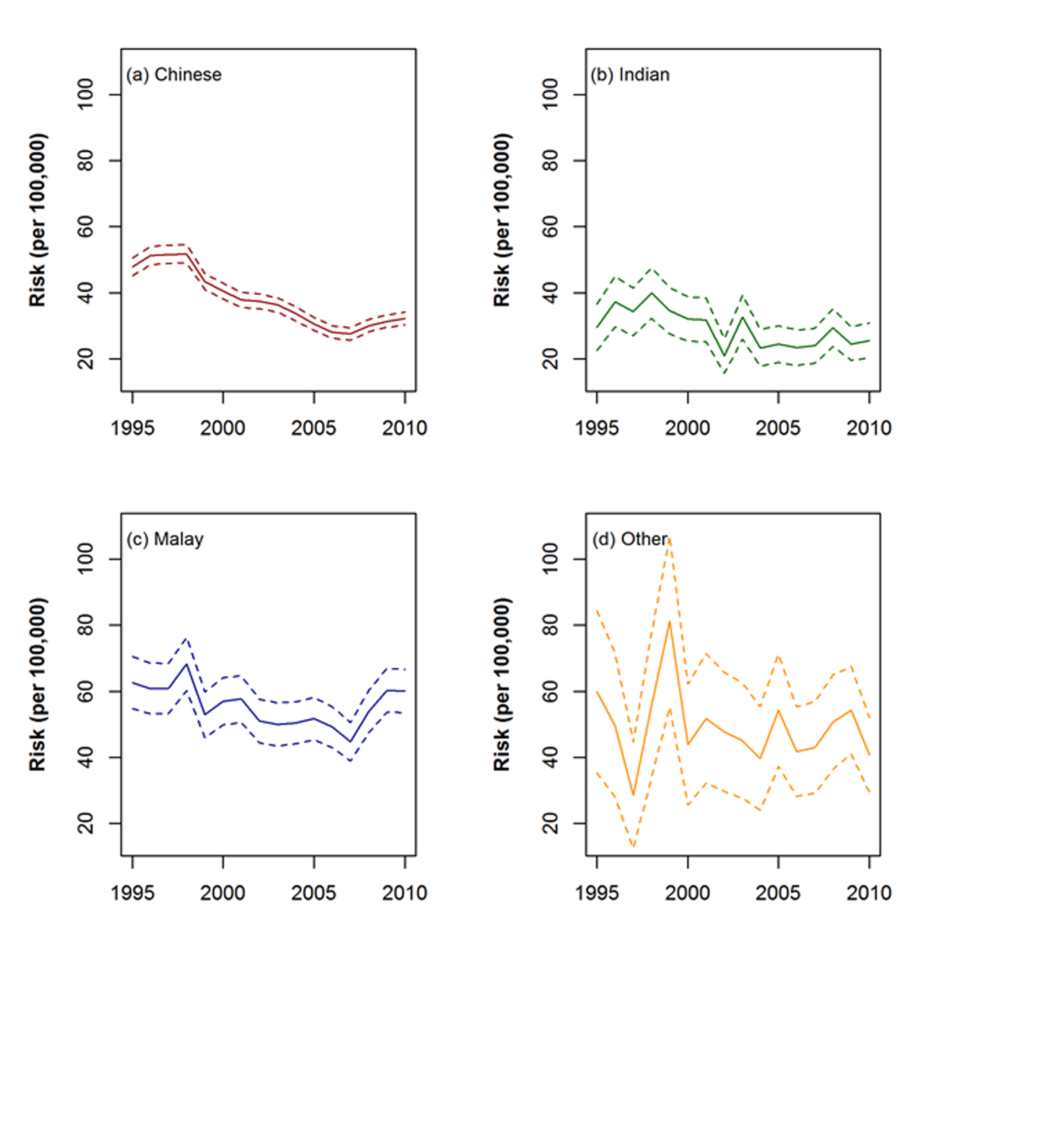

Supplement: Supplementary file 1 — Additional file 1: Figure S1: Age-standardized incidence rate of tuberculosis, stratified by ethnicity. (TIFF 1010 KB) [file 12889_2014_7217_MOESM1_ESM.tiff]

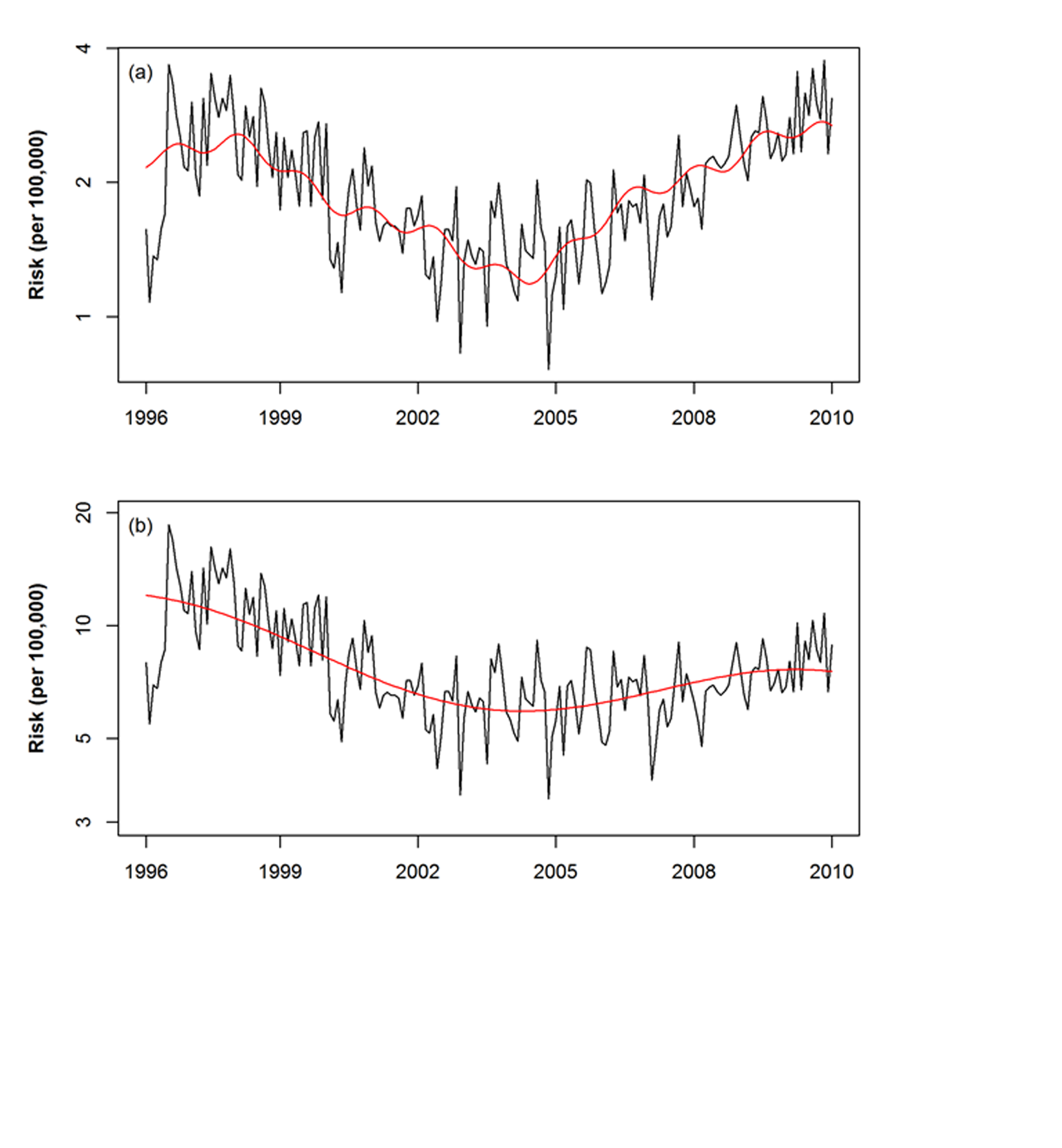

Supplement: Supplementary file 2 — Additional file 2: Figure S2: Trend model of monthly TB risk (a) residents (b) non-residents. (TIFF 882 KB) [file 12889_2014_7217_MOESM2_ESM.tiff]

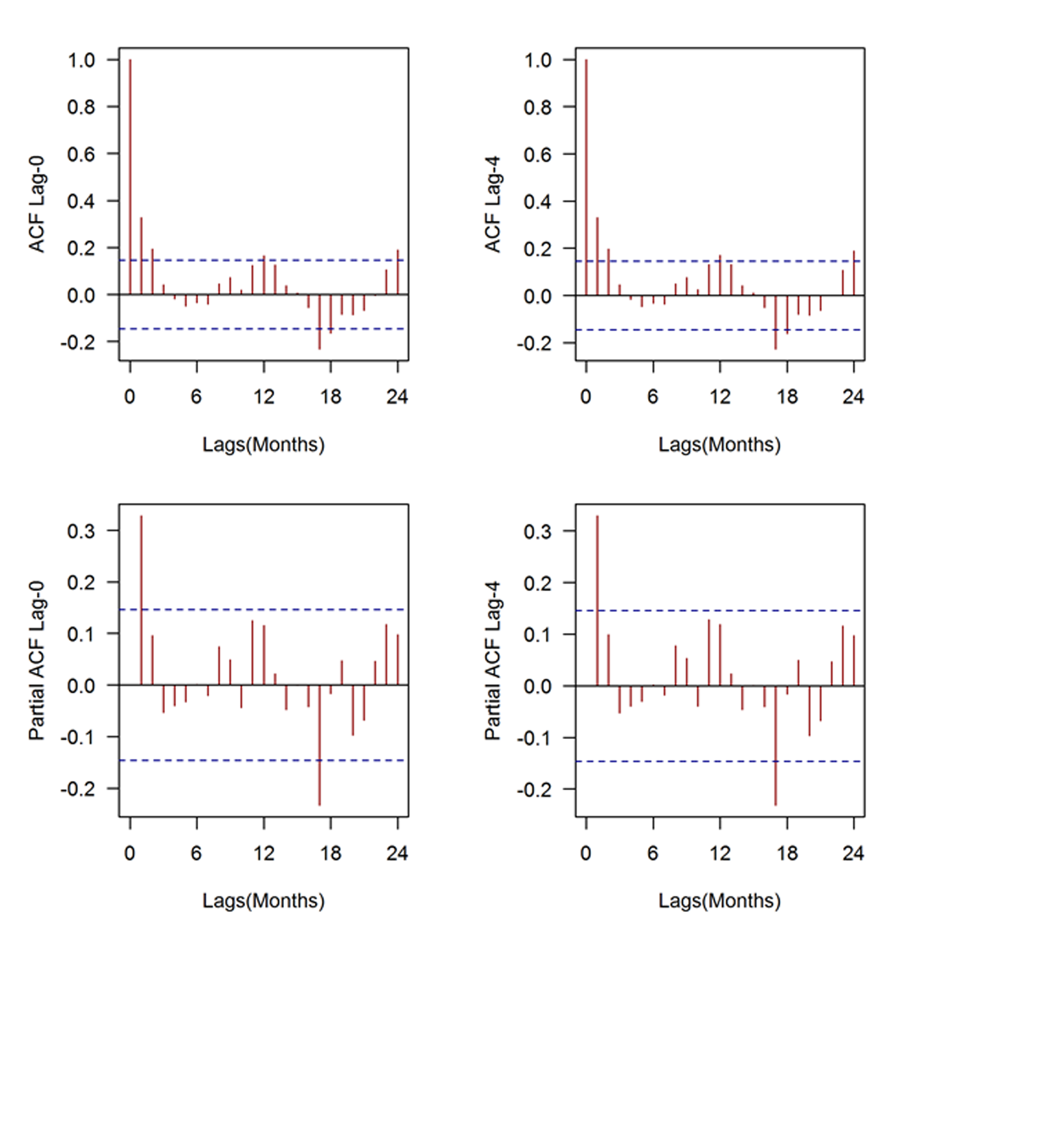

Supplement: Supplementary file 3 — Additional file 3: Figure S3: Autocorrelation (ACF) and partial autocorrelation (PACF) plots of fitted residuals (Residents). (TIFF 859 KB) [file 12889_2014_7217_MOESM3_ESM.tiff]

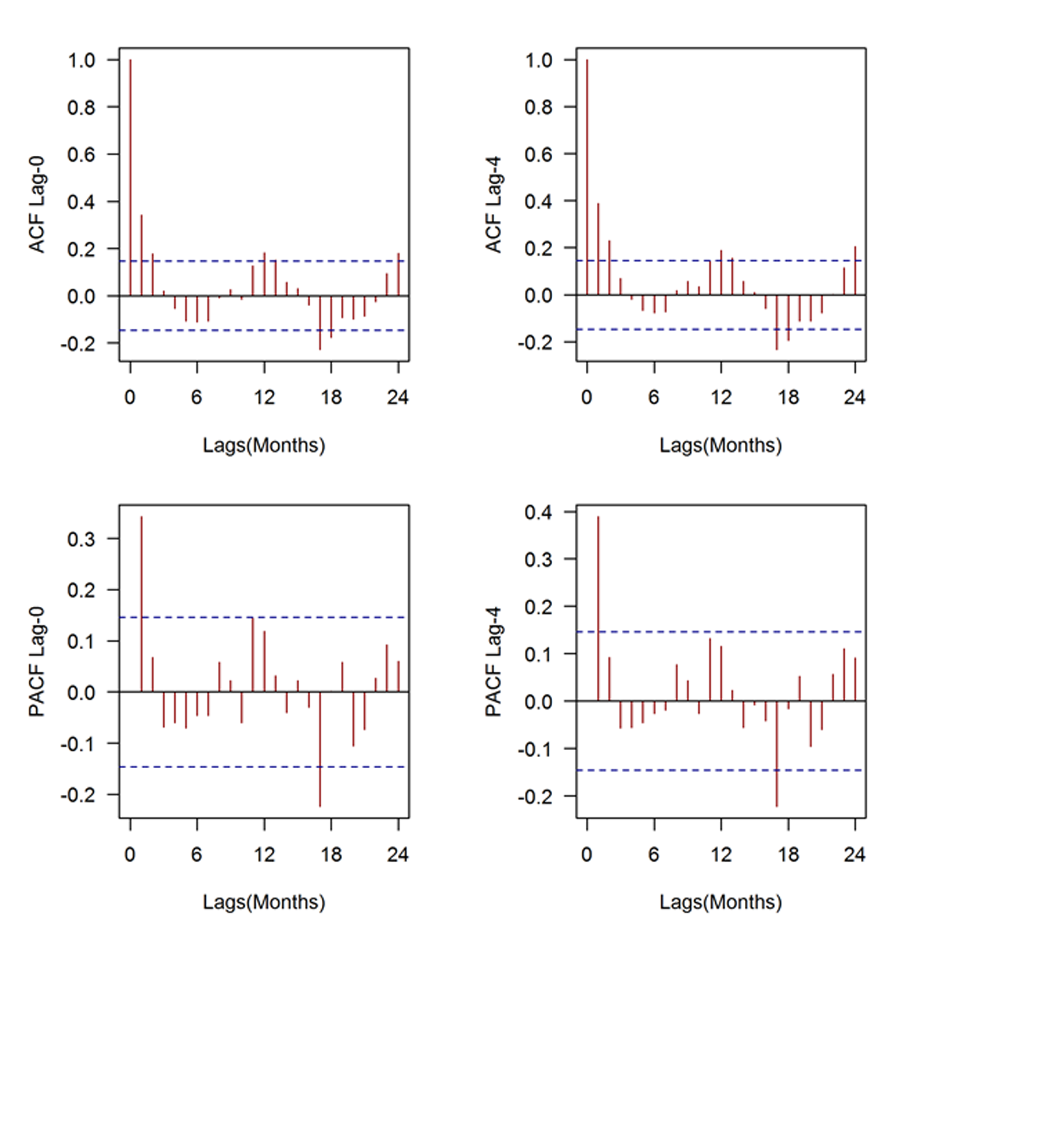

Supplement: Supplementary file 4 — Additional file 4: Figure S4: Autocorrelation (ACF) and partial autocorrelation (PACF) plots of fitted residuals (Non-residents). (TIFF 858 KB) [file 12889_2014_7217_MOESM4_ESM.tiff]

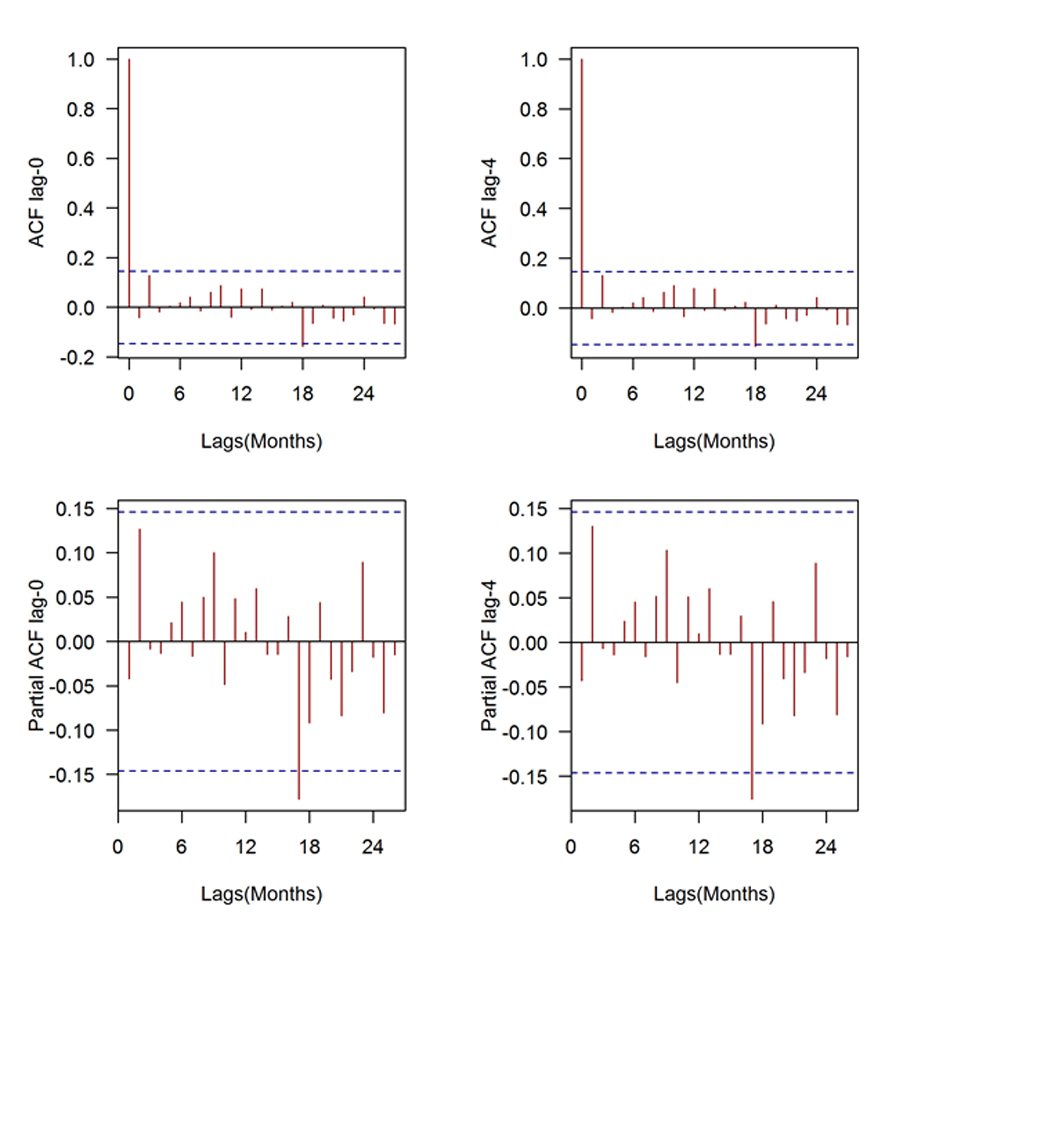

Supplement: Supplementary file 5 — Additional file 5: Figure S5: Autocorrelation and partial autocorrelation of final SARIMA model residuals (Residents). (TIFF 888 KB) [file 12889_2014_7217_MOESM5_ESM.tiff]

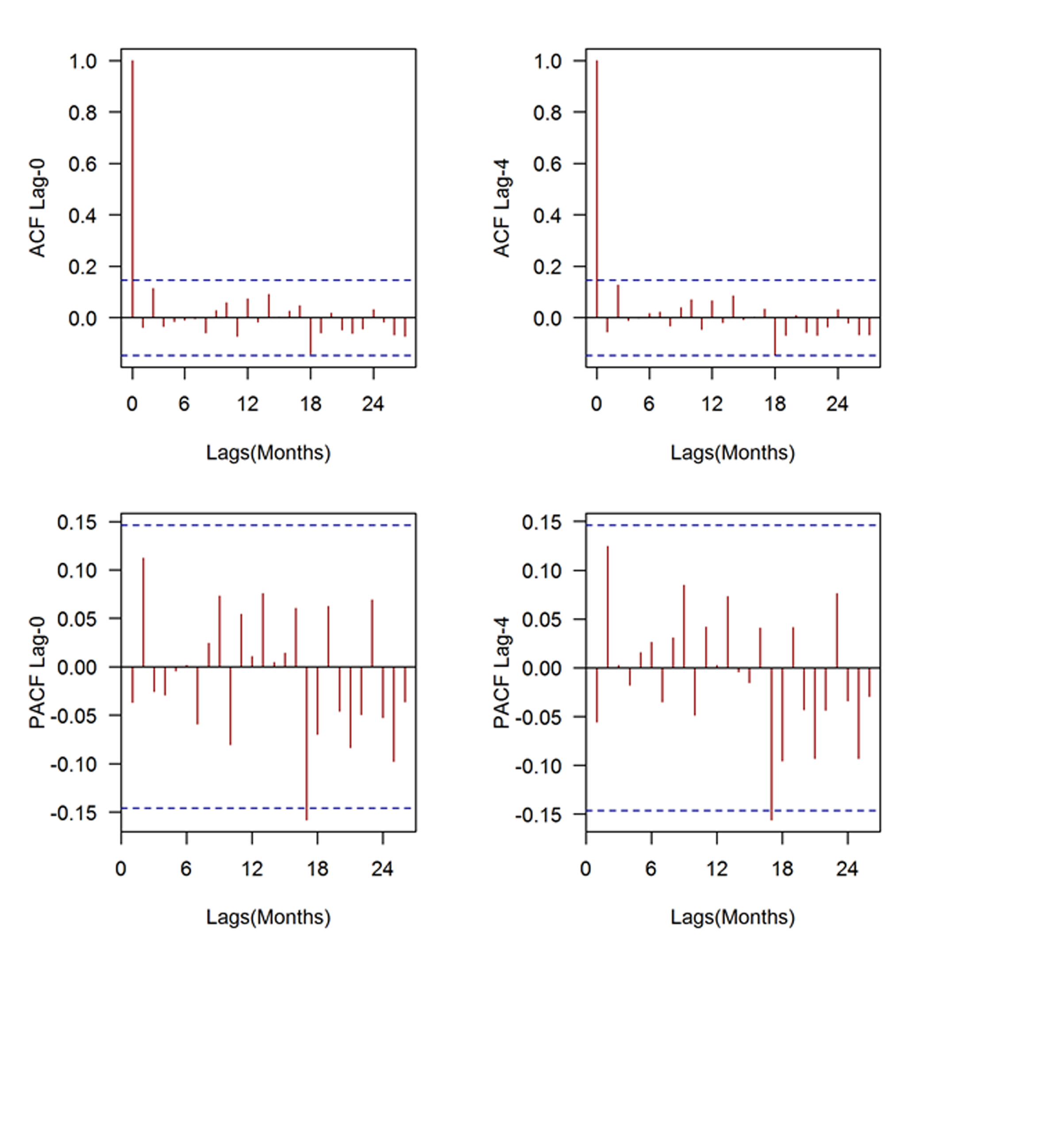

Supplement: Supplementary file 6 — Additional file 6: Figure S6: Autocorrelation and partial autocorrelation of final SARIMA model residuals (Non-residents). (TIFF 898 KB) [file 12889_2014_7217_MOESM6_ESM.tiff]
